# The Case of Dr. Beale

**Published:** 1854-12

**Authors:** 


### THE CASE OF DR. BEALE.

Dr. Beale, a dentist of Philadelphia, was convicted, a short time since, of the crime of rape upon a young lady while in his operating chair and under the influence of sulphuric ether, and has been sentenced to the penitentiary. The only testimony in the case was that of the young lady herself, who professed, though wholly powerless from the effects of the anesthetic, to be conscious of all that was transpiring and sensible to all external impressions. We must say that the truth of the charge strikes us as eminently improbable. That the subject should be so perfectly deprived of certain faculties by the ether, and yet so fully possessed of others; that such an act should be performed at all, in such a position, and under such circumstances, to us seems wholly incredible. It appears to us that a man has seldom been condemned to ignominious punishment upon more slender evidence; and Dr. Beale was a man whose character, up to the time of the unhappy charge, was without reproach. Numerous petitions have been sent to the Governor of Pennsylvania for the pardon of the prisoner, among the rest, by the Dentists of New York, who have investigated the facts of the case and arrived at the conclusion, that he is guiltless of the crime alleged against him. We hope he will be pardoned.

